# Safety, feasibility, and effectiveness of a novel spray cryotherapy technique in a canine model

**DOI:** 10.1002/ctm2.315

**Published:** 2021-02-01

**Authors:** Hongxia Duan, Xuan Li, Xuan Long, Xinyang Liu, Shuanshuan Xie, Changhui Wang

**Affiliations:** ^1^ Department of Respiratory Medicine, Shanghai Tenth People's Hospital Tongji University School of Medicine Shanghai China

Dear Editor

Spray cryotherapy (SCT) is an energy‐based technique which employs freezing to destroy undesired tissues. We report the impact of SCT treatment on bronchi mucosal surface of canines in China firstly.

Twelve labradors underwent SCT and were assessed for the basic vital signs and health status prior to and post‐SCT. At the time of post‐SCT 0 day, 2 days, 7 days, 30 days, 60 days, and 90 days, safety and histological changes were observed. The SCT device (Figure 1A) was provided by Senscure Biotechnology Co., Ltd (Ningbo, China). This system provides liquid nitrogen at 25 psi through a flexible catheter (Figure 1B) with a 16‐hole nozzle (Figure 1C), in which nitrogen is forced out by breathing after the spray, the surface temperature of the treated bronchi can be rapidly reduced to −110∼−196°C. The system freezes a circular target area of 3–8 mm in length and 0.02–0.5 mm in depth by adjusting the flow rate, volume of liquid nitrogen spray, and the duration of spraying.

**FIGURE 1:**
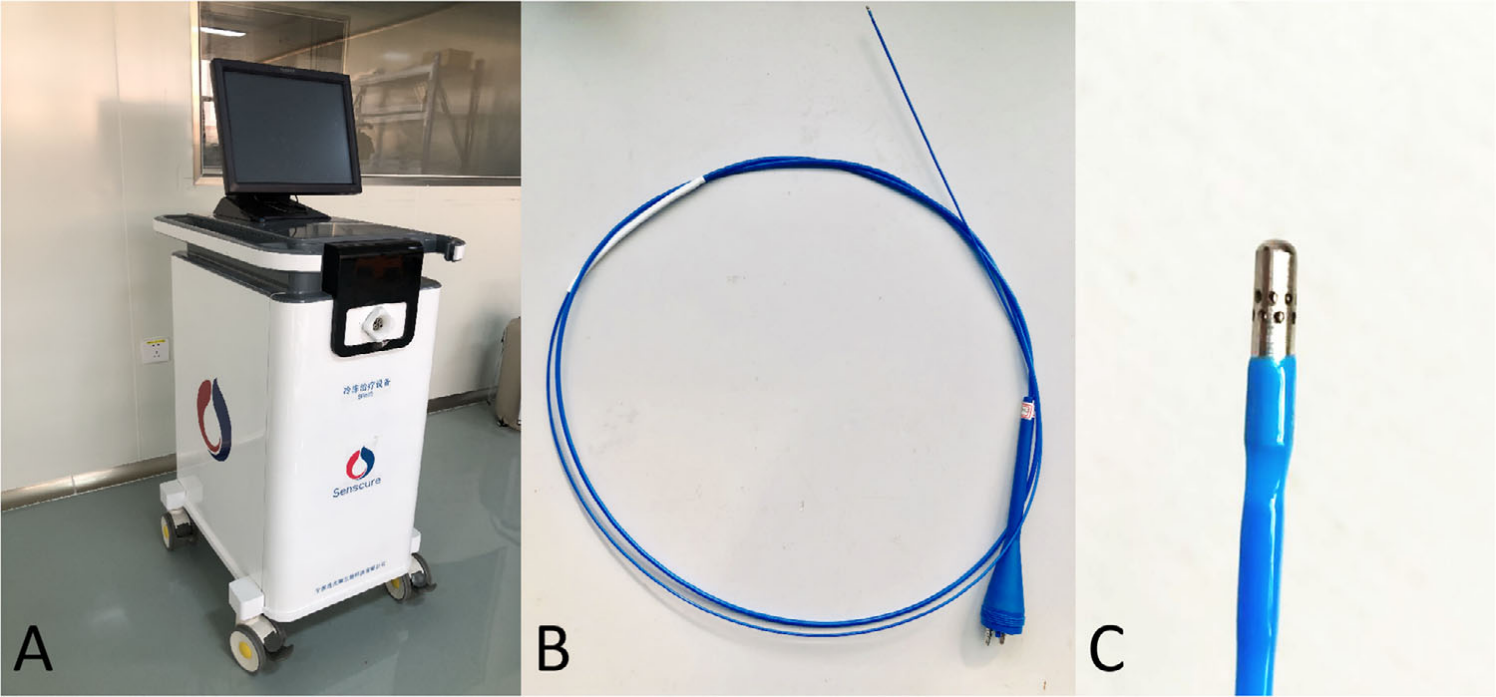
The liquid nitrogen spray device. (A) Liquid nitrogen storage and control panel. (B) The liquid nitrogen pipeline is made of polymer material and insulated by a vacuum layer. (C) The spray nozzle of liquid nitrogen, with two rows of eight holes arranged around, can spray liquid nitrogen evenly throughout the bronchi

The results showed that SCT is feasible and safe in canine bronchi. Once SCT was initiated, ice crystal formation was observed on the probe (Figure 2A) and gradually adhered to the bronchial wall (Figure 2B), and finally embraced the bronchial wall at the end of freezing (Figure 2C). After thawing, mild mucosal hyperemia was seen in the airways (Figure 2D). No active hemorrhage occurred in any animal. In cryo‐day 2 group, mild hyperemia was observed in all treated bronchi (Figure 2E). Seven days later (Figure 2F), including assessments in cryo‐day 30, cryo‐day 60, and cryo‐day 90 group (Figure 2G), the airway appeared essentially normal (Figure 2H) with no hyperemia, edema, stenosis, and scarring. During the experiment, no serious anesthesia and procedure‐related complication was observed, and no death occurred, indicating that the use of SCT is safe in the lungs of canines, which is consistent with previous research.^1^

**FIGURE 2:**
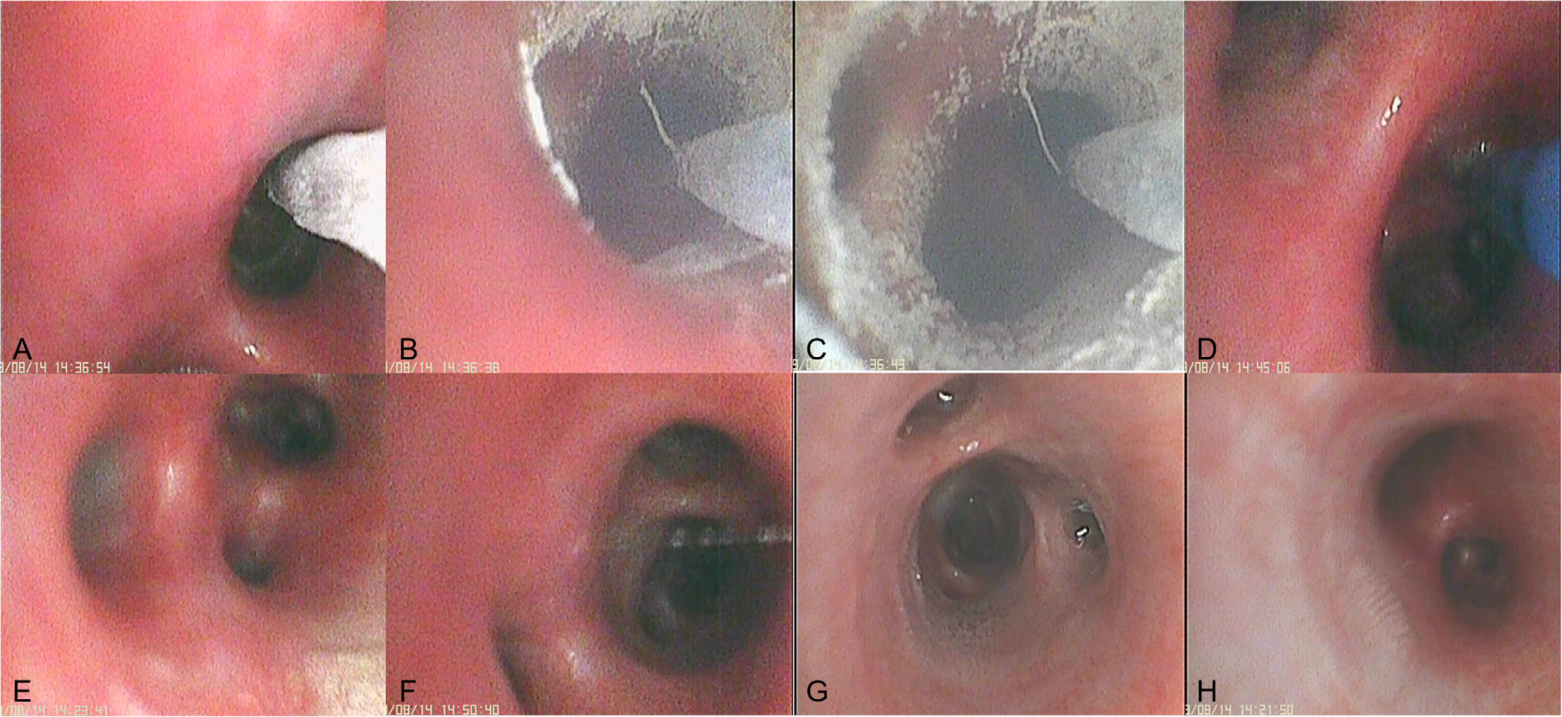
Bronchoscopic evaluation of bronchi after SCT. (A) Pre‐SCT, there was no abnormality in the bronchial mucosa. (B) and (C) During SCT, the bronchial mucosa was gradually frozen, and vapor crystallized on the surface of the bronchial mucosa. (D) Mild hyperemia was observed in the bronchial mucosa immediately after SCT. (E) Mild hyperemia in the mucosa was observed by bronchoscopy 2 days after SCT. (F) Seven days after SCT. (G) The bronchi mucosa restored normal under bronchoscopy 30 days after SCT. (H) Normal mucosa under bronchoscopy. Abbreviation: SCT, spray cryotherapy

Besides, SCT regenerates epithelial cells with no scar formation. As shown in Figure 3, epithelium histology restores normal within 7 days post‐SCT. Compared with the normal group (Figure 3A), the epithelium disappeared, with mild edema and a small number red blood cells and inflammatory cells seen in the submucosa; no obvious change was observed in the smooth muscle layer in the bronchial tissues in cryo‐day 0 group (Figure 3B). Two days later, the epithelium began to recover, no obvious goblet cells were found, and local epithelial cells were slightly loose histologically; there was still mild congestion and edema in the submucosa with no scar formation in the smooth muscle layer (Figure 3C). Seven days later (Figure 3D), the tissue recovery is about the same as normal tissue and normal structures. The assessment of 90 days post‐SCT is shown in Figure 3E.

**FIGURE 3:**
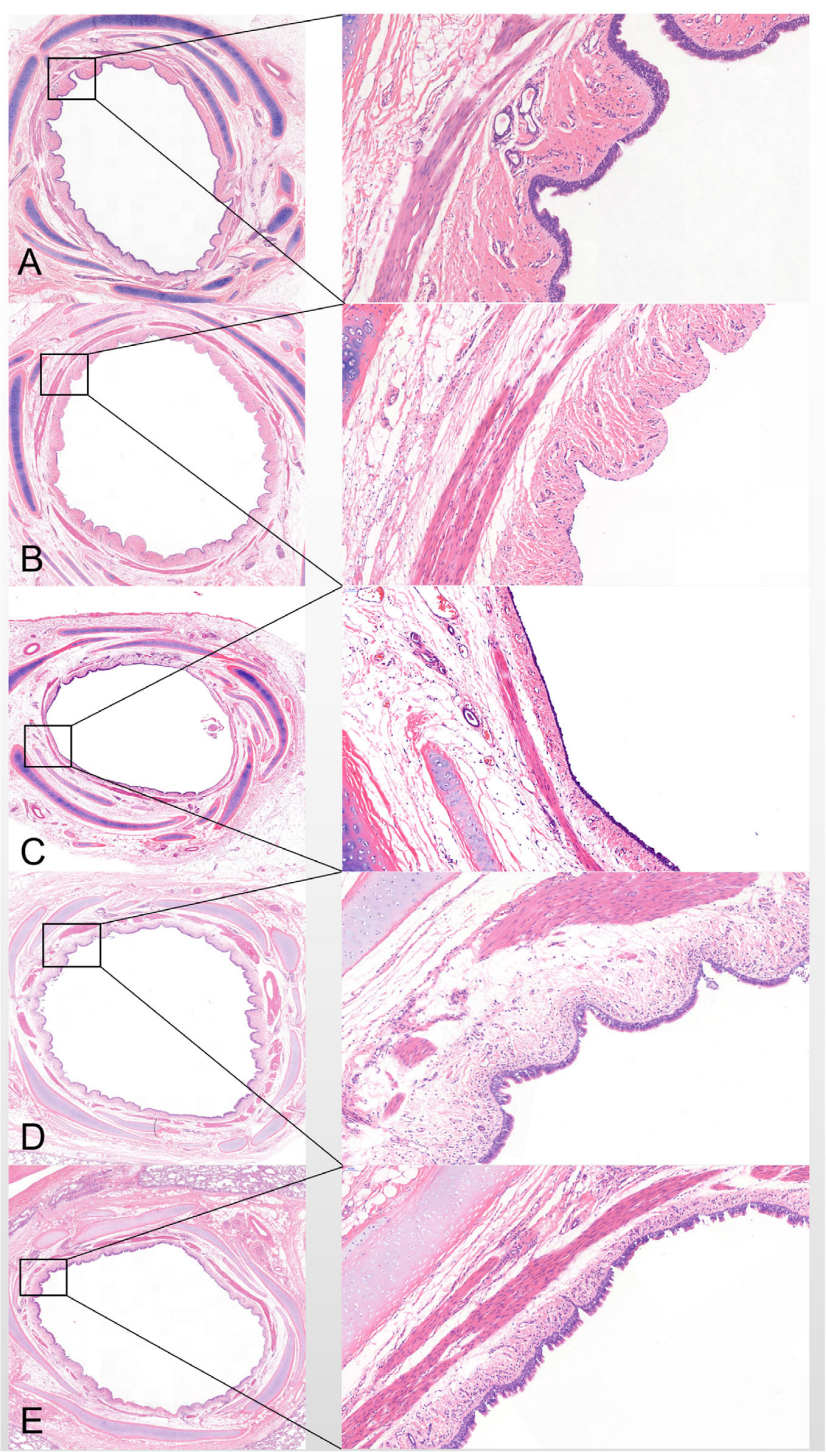
HE staining of bronchial wound healing after SCT. (A) Pathology of the normal bronchus, which was composed of the complete mucosa, submucosa, smooth muscle, and cartilage layer. (B) Pathology of the bronchus after immediate SCT, showing loss of the bronchial mucosa as the most obvious pathological feature. (C) Bronchial epithelial cells were sparse and incomplete 2 days after SCT, but they tended to become normal as compared with (B). (D) Epithelial cells were arranged neatly and completely, and became almost normal 7 days after SCT. (E) The bronchus restored normal 30 days after SCT. The structures of the submucosa, smooth muscle and cartilage were intact in all sections. (F) Bronchial smooth muscle thickness was obtained by the mean of all specimens measured by Image‐Pro Plus 6.0. The smooth muscle thickness decreased immediately after SCT (109.4 ± 10.53 vs 144.2 ± 7.034 mm, p = 0.0185), and 2 days later the smooth muscle thickness became normal and remained at a normal level within the 90‐day observational period. The pathological images on the left are all magnified 2.5 ×, and those on the right are magnified 20 ×. Bars represent the mean ± SD of bronchial smooth muscle thickness in seven separate groups. Abbreviation: SCT, spray cryotherapy

Our experience and practice in safe use of SCT are summarized as follows. Firstly, anesthetics that may strongly inhibit the central respiratory function should be avoided. Zoletil50 is currently a relatively safe anesthetic for use in animals because it can quickly relax muscles and eliminate superficial pain and visceral sensation without inhibiting the respiratory center, which is extremely important for perioperative safety. Second, construction of artificial airway is very necessary for safety, because the artificial airway is conducive to the rapid exhalation of the large amount of nitrogen and can reduce the adverse effect of low‐temperature gas on cardiopulmonary function. Thirdly, it is also crucial to understand and prevent against the effect of cold stimulation on cardiopulmonary function, knowing that hypoxemia, sympathetic nervous, and vagal stimulation occurring during cold stimulation may be a trigger of arrhythmia, which is a potential hazard for sudden death.^2^, ^3^, ^4^ Thus, vital signs should be monitor meticulously during the whole process of surgery, and the backup of ventilator and pacemaker is necessary. According to our experience, it is necessary to maintain at least a 1‐minute interval of airway patency between two spray cryotherapies. Lastly, the right middle lobe is located in the middle of the upper right lobe and the lower right lobe and has a small lung volume anatomically, which makes SCT more risky. In addition, due to the its proximity to the heart's sinoatrial node, vagus nerve and sympathetic nerve, in order to avoid the occurrence of arrhythmia, the right middle lobe of the lung is not the SCT target.

The other aim of our study is to observe the histopathological changes of bronchi in the repair process after SCT. It was found that cytokeratin five positive basal cells survived after SCT (Figure S1), it promoted rapid differentiation, proliferation, and re‐epithelialization of basal cells after epithelial damage and wound healing without scar formation. As a result, HE staining showed a significant regeneration of epithelial cells 2 days after SCT.

To conclude, this is the first study in China to use SCT in a canine model for a long‐time systematic observation. SCT has the characteristics of rapid and maneuverable ablation of the epithelium without causing scar formation. It causes little damage to basal cells under the epithelium and can activate epithelial stem cells in the early stage of injury and help heal the wound rapidly. The data obtained in this study may provide a novel insight into and laid a theoretical basis for interventional therapy of airway disease. Necrosis of epithelial cells provides an opportunity to induce re‐epithelialization and normalization of respiratory mucosal epithelial tissues. Taken together, these preclinical data pave the way for SCT study in airway diseases.

## Supporting information

Supporting Information
