# Supplementary figures and images for: Safety, feasibility, and effectiveness of a novel spray cryotherapy technique in a canine model
Source: Clin Transl Med. 2021 Feb 1;11(2):e315. doi: 10.1002/ctm2.315 (PMC7851571; doi:10.1002/ctm2.315)

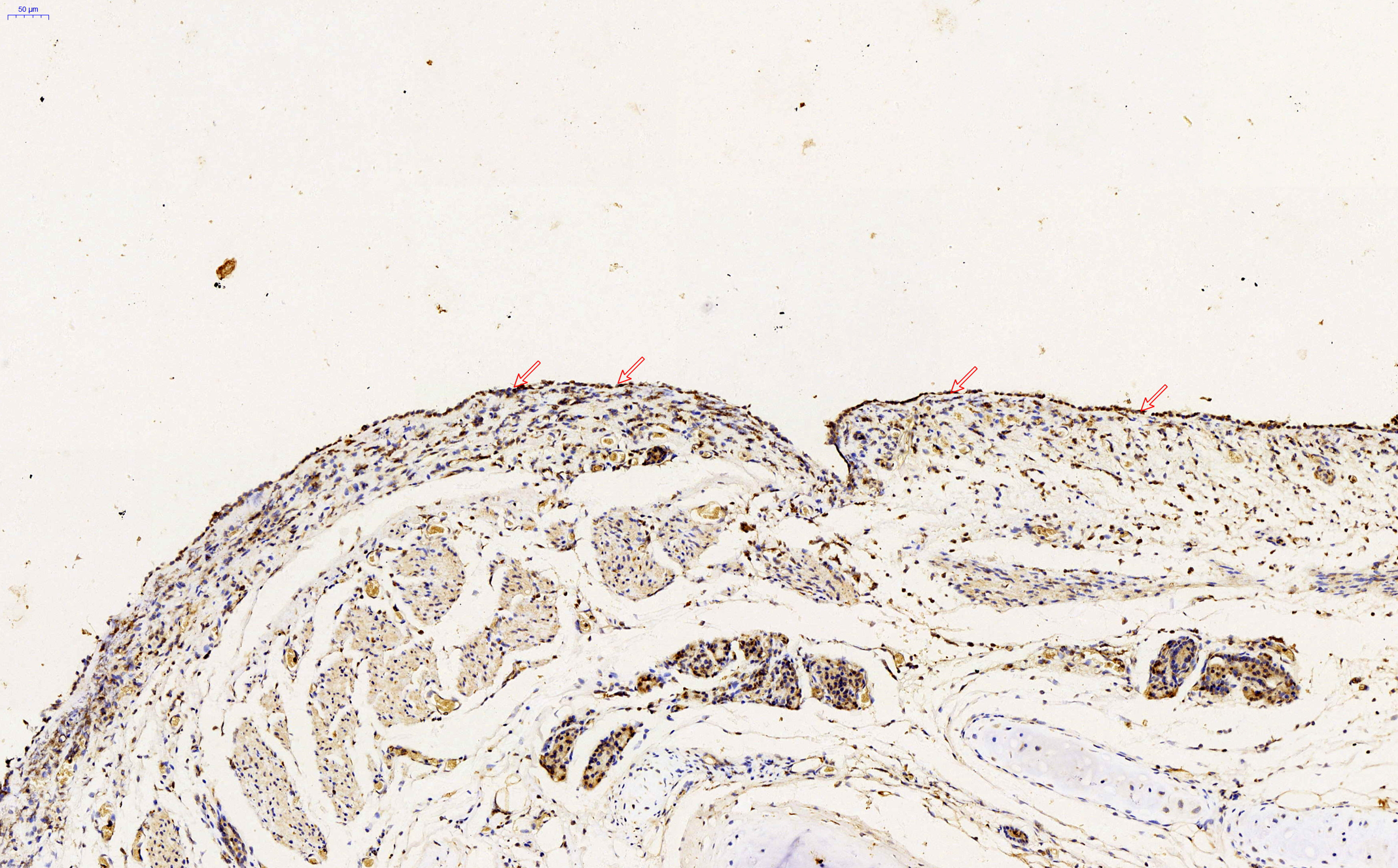

Supplement: Supplementary file 1 — Supporting Information [file CTM2-11-e315-s001.tif]
